# Special Issue on “Cellular and Molecular Mechanisms Underlying the Pathogenesis of Hepatic Fibrosis II”

**DOI:** 10.3390/cells11152403

**Published:** 2022-08-04

**Authors:** Ralf Weiskirchen

**Affiliations:** Institute of Molecular Pathobiochemistry, Experimental Gene Therapy and Clinical Chemistry (IFMPEGKC), RWTH University Hospital Aachen, D-52074 Aachen, Germany; rweiskirchen@ukaachen.de; Tel.: +49-(0)241-8088683

## 1. Introduction

**Figure 1:**
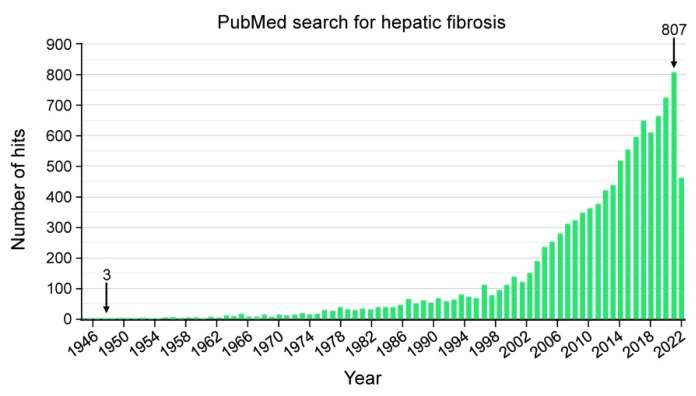
PubMed search for the query “hepatic fibrosis”. This example search documents that this research area has found increasing interest during the last decades. Whereas with the search term “hepatic fibrosis” only 3 hits can be found for the year 1948, there were already 807 entries in the year 2021. Especially after the turn of the millennium, the number of publications in this field has increased dramatically. Please note that the year 2022 only covers the time interval from January to the end of July.

Hepatic fibrosis is a common type of liver disease that is attracting increasing attention of basic scientists and clinicians worldwide. This is best documented in a simple PubMed search. When searching this database with the search terms “liver fibrosis” or “hepatic fibrosis”, one will find a total of 20,173 or 10,048 entries in the 34 million citations listed on the database (search conducted on 31 July 2022). Notably, the number of articles dealing with the respective topics permanently increased from year to year (Figure 1).

A recent estimate that is based on population-based studies and screening for liver fibrosis with noninvasive tests, transient elastography, and radiological methods has shown that the overall percentage of subjects suffering from liver fibrosis is around 5% in the general population and about 18% to 27% in populations with risk factors for liver disease [1]. Although the molecular mechanisms and pathways, including the soluble mediators that drive hepatic fibrosis, are very well studied and several promising drugs are being tested in advanced clinical trials, there is still no approved therapy [2,3]. Moreover, the list of unmet needs that was recently published for this research area was hard-hitting, showing that there is still an intensive lack of knowledge in many sub-areas of this research field, including the basic principles, etiology, therapy, assessment, discovery of anti-fibrotic targets, and the conduct of meaningful clinical studies [4].

In this Special Issue, 158 international experts have compiled 11 reviews and 10 originals in which novel findings and concepts in experimental and clinical liver fibrosis research are presented. Covering a wide range of aspects, the compilation of articles demonstrates that scientists and clinicians intensively try to address the mentioned gaps and unmet needs in the establishment of effective antifibrotic therapies.

This Special Issue is a continuation of a previous set of *Cells* articles that were published at the beginning of 2020. Like the former article collection, the follow-up contains a curated collection of cutting-edge peer-reviewed reviews and originals, reporting on novel aspects of etiology, cellular and molecular drivers, the discovery of antifibrotic targets, innovative therapeutic strategies, and factors contributing to the pathogenesis and resolution of hepatic fibrosis. The contributions show that the research in this area is very vibrant, giving hope that the increasing knowledge will allow us to establish the urgently needed breakthrough therapies for liver fibrosis in the near future.

## 2. Update on Etiological Causing Liver Fibrosis

Khanam and colleagues summarized the current knowledge of cellular and molecular mechanisms involved in the progress of hepatic fibrosis. The authors discuss the different etiologies of hepatic fibrosis (i.e., viruses, alcohol, fat, and autoimmune diseases) and present concise information about the relationship of the different liver cells in driving the expression and deposition of excess extracellular matrix (ECM), which is the hallmark of fibrosis. Moreover, exciting details on how exosomes, apoptotic bodies, inflammasomes, and microRNAs (miRs) can impact the outcome of hepatic fibrosis are presented [5].

The review of Delgado et al. shed light on the biology of hepatic stellate cells (HSCs) that are in the disease liver the major cellular contributors to excess ECM deposition. In special focus of this contribution are ribonucleic acid (RNA)-binding proteins and other RNA-regulators that significantly contribute to the activation and metabolic reprogramming of HSCs during progression of hepatic fibrogenesis [6]. This article illustrates that the activity of these molecular factors is highly dynamic, and subtle disturbances in their proper regulation result in dysregulated protein expression and altered functions impacting the general process of fibrogenesis. As such, the elucidation of these proteins might help to identify novel therapeutic targets for attenuation or reversal of liver fibrosis.

The complex alterations occurring in HSC activation during progress of hepatic fibrosis are also discussed by Smith-Cortinez and colleagues [7]. In their article, novel data are presented showing that the activation process of HSC is marked by a simultaneous induction of glycolysis and mitochondrial metabolism, as indicated by the increased activity of rate-limiting enzymes of these pathways, occurrence of extensive mitochondrial fusion, and increased oxidative phosphorylation. The authors conclude that these alterations are necessary to meet the high-energy demands associated with induced cell proliferation, migration, contraction, and ECM production in HSC during the pathogenesis of fibrosis. In line with this assumption, the authors could demonstrate that the inhibition of glycolysis potently suppressed the activation process suggesting that targeting mitochondrial respiration might offer novel molecular targets to block ongoing fibrosis.

The contribution of Lafoz and colleagues summarizes the current knowledge on liver sinusoidal endothelial cells (LSECs) biology during liver injury and repair. Loss of fenestrae, altered vasodilatory features, and drops in anti-inflammatory, anti-thrombotic, anti-angiogenic, anti-fibrotic, and regenerative capacities are the most important alterations in LSECs occurring during progress of hepatic fibrosis [8]. In contrast, LSEC are also important cellular contributors in liver regeneration and fibrosis resolution by coordinating the recruitment and activity of various immune cells and orchestrating the regenerative process. Consequently, the authors conclude that the unique structural, mechanical, and biological properties of LSEC might offer a large variety for therapeutic interventions.

## 3. Novel Molecular Modulators of Hepatic Fibrosis

Kim et al. unraveled the molecular mechanisms by which the death-associated protein 6 (Daxx) alleviates TGF-β-induced hepatocyte epithelial-to-mesenchymal transition. Daxx is a multifunctional protein that regulates a wide range of biological processes and resides in multiple locations in the nucleus and in the cytoplasm [9]. The authors provide a large wealth of experimental data showing that Daxx can alleviate hepatic fibrosis by the inhibition of Smad2 acetylation and phosphorylation. In line with the proposed therapeutic activity of Daxx, the overexpression of Daxx allowed the attenuation of thioacetamide-induced liver fibrosis in mice [9].

Geervliet and Bansal present a comprehensive overview of the role and expression of matrix metalloproteinases (MMPs) in liver diseases [10]. These enzymes are a class of calcium-dependent zinc-containing endoproteinases that can degrade ECM components in normal liver. However, on the contrary, dysregulation of individual MMPs can also contribute to the progression of liver disease, showing the complex activity of these proteins. The different facets of the enzymes are well discussed in the article. It also highlights promising experimental studies in which the balance between ECM deposition and degradation was successfully restored by targeting different MMPs or their inhibitors. Nevertheless, none of these findings were translated yet into clinical trials, but there are examples in which MMPs or MMP genotype polymorphisms were reported as suitable biomarkers to estimate the risk for hepatic diseases [10].

In the contribution by Schippers et al., the effects of the intestinal alkaline phosphatase (AP) was studied in mice with carbon tetrachloride-induced liver fibrosis. This enzyme has the ability to detoxify bacterial lipopolysaccharides (LPS) by removing a phosphate group from its lipid A moiety, which prevents its recognition by toll-like receptor 4. The authors could demonstrate that the di-phosphoryl lipid A strongly stimulated fibrogenic and inflammatory activities in primary rat HSC and RAW264.7 macrophages, while the mono-phosphoryl lipid A were unable to provoke these effects [11]. Moreover, the systemic administration of exogenous intestinal AP in fibrotic mice resulted in reduced macrophage infiltration and lowered activation of HSC, as assessed by reduced expression of desmin and α-smooth muscle actin. Although the overall content of hepatic collagen content was unaffected by this treatment, the study demonstrates that LPS dephosphorylation by intestinal AP might protect the liver from biologically active gut-derived LPS.

## 4. Epigenetics in Liver Fibrosis

Actually, there is an intense discussion on the impact of DNA methylation, histone modifications, and non-coding RNAs for the pathogenesis of hepatic fibrosis. The review by Claveria-Cabello and coworkers provides an up-to-date source for information about how these epigenetic factors contribute to the activity of HSC. In particular, information is provided on histone deacetylases (HDACs), which enzymatically remove acetyl groups from acetylated lysine residues, thereby restoring the positive electrical charge of this amino acid, resulting in a higher affinity for negatively charged DNA and a general silencing of gene expression. Furthermore, this reference article highlights the mechanisms by which different HDAC inhibitors exert their anti-fibrotic effects through modulating the chromatin topology and activity of non-histone proteins, such as transcription factors. In sum, the article demonstrates that strategies for the modulation of histones hold promise for the development of new therapies for liver fibrosis but that further work is required to unravel the epigenetic events and their crosstalk to dissect and understand their precise role in HSC activation [12].

## 5. Nonalcoholic Fatty Liver Disease and Nonalcoholic Steatohepatitis

Most articles in this Special Issue focus on aspects of nonalcoholic fatty liver disease (NAFLD) and nonalcoholic steatohepatitis (NASH), which are terms for conditions caused by excess hepatic fat. The complexity of lipid diversity and dynamics during diseases associated with surplus fat buildups is illustrated by Molenaar and coworkers [13]. Their review provides expert knowledge on mechanisms by which nutritional overload from exogenous sources and the endogenous overproduction of lipids leads to parenchymal fat accumulation. They also discuss how important risk factors for hepatic steatosis, such as the common Ile148Met variant of the patatin-like phospholipase domain-containing protein 3 (PNPLA3), contribute to hepatic lipid accumulation and how the promotion of lipid catabolism and secretion can be therapeutically effective. The reading of this article is an ideal introduction for the article by Schwartz and colleagues, deepening this theme. In this study, a clinical compound library was screened and the JAK1/JAK2/ACVR1 inhibitor momelotinib identified as a potent inhibitor of *PNPLA3* mRNA expression in primary human hepatocytes and HSC, a *Pnpla3*-synchronized mouse model, and in a novel mixed lineage 3D spheroid model, consisting of HepG2 and HSC cell line LX-2 [14]. Mechanistically, the authors could demonstrate that momelotinib conveys its therapeutic effects by reducing the expression of profibrotic and proinflammatory markers, lowering the chromatin accessibility at the *PNPLA3* locus, and further by inhibition by the BMP signaling pathway [14].

Drescher and colleagues investigated the functional role of the cell adhesion molecule L-Selectin/CD62L in two mouse models of steatohepatitis and human NASH. This protein is found on the cell surface of leukocytes, playing an important role in both the innate and adaptive immune responses. The authors could show that the livers of mice lacking CD62L or treated with a therapeutically effective anti-CD62L blocking antibody were protected from diet-induced steatohepatitis, exhibited an enhanced hepatic infiltration of regulatory T cells, and showed a strong anti-oxidative stress response [15]. In addition, CD62L expression in human NASH correlated with the disease activity, suggesting this protein as a promising target for NAFLD therapies.

Similar findings were reported by Heinrichs et al. for the macrophage migration inhibitory factor (MIF). This pleiotropic cytokine is released from T cells and macrophages and counteracts anti-inflammatory actions by regulating factors involved in immune responses. The authors report that hepatic *Mif* expression was strongly induced during NASH in mice and men, while *Mif*^−/−^ mice displayed reduced more liver fibrosis than wild type mice after being subjected to a NASH-inducing diet. The major source of MIF in the liver was noticed in hepatocytes and line-specific deletion of *Mif* in hepatocytes resulted in reduced NASH-mediated liver fibrosis in a degree similar to those observed in constitutive *Mif*-deficient mice [16]. The authors could further demonstrate that MIF had a direct effect on natural killer T cell polarization, thereby modulating the intrahepatic micro-environment during NASH. In sum, this article demonstrates that, similar to PNPLA3 and CD62L, MIF might be an attractive target to interfere with inflammation and fibrosis during NASH.

Other new findings on NAFLD modulators were highlighted in a review by Lin et al. in which the biological functions of critical miRs implicated in the pathogenesis of liver disease are discussed. The best characterized one, i.e., miR-29a, acts protectively during liver injury by interfering with the activity of DNA methyltransferases, reducing oxidative stress and inflammation, modulating mitochondrial metabolisms, and by reprogramming hepatic lipid distribution. It further impacts innate immune response and acts antifibrotic by downregulation of collagen expression and inducing HSC apoptosis. However, some of these activities are still unraveled or controversially discussed, showing the necessity of additional studies investigating the precise functions of miR-29a in different settings of NAFLD and NASH [17].

Marokovic and colleagues discuss the relevance of another miR. They discuss that miR-221 is upregulated in many models of liver fibrosis, while its suppression inhibits the expression of fibrogenic gene signatures, suggesting this miR as a good biomarker for assessment of hepatic fibrosis. The article provides an excellent overview about the biology of this noncoding RNA molecule, including biogenesis, regulation, biological activity, diagnostic relevance, and its potential therapeutic capabilities, in mitigating hepatic fibrosis or other liver disease complications, such as hepatocellular carcinoma. In addition, the authors provide a well thought out list of unanswered questions that need to be solved before introducing miR-21 in a clinical setting [18].

Asimakopoulou identified the lipid droplet coating protein Perilipin 5 (PLIN5) as a critical regulator of liver homeostasis in NASH by comparatively analyzing the pathogenesis of liver damage in wild type and *Plin5*^-/-^ mice. In this study, the loss of PLIN5 was associated with reduced hepatic damage and fat, differences in lipid metabolism, mitigated activation of inflammasome activity, and reduced levels of inflammatory mediators in a high-fat diet model [19]. Based on these findings, the authors suggest that the blocking of the PLIN5 expression could have therapeutic implications. In line, the same group demonstrated that the loss of PLIN5 prevents TGF-β-induced activation of HSC and ECM production by blunting profibrogenic TGF-β signaling, SNAIL activity, and STAT3 activation [20]. In conformity, the expression of fibrosis-associated marker genes, including Fibronectin, collagen type I, Vimentin, Desmin, and α-smooth muscle actin, showed higher expression in cultured HSC isolated from *Plin5*^-/-^ mice compared to those isolated from wild type mice, while the restoration of PLIN5 expression reduced their expression [20]. Although more research on the biological activities of PLIN5 is still required, both studies equally suggest PLIN5 as a novel potential therapeutic target for NAFLD/NASH therapy.

## 6. New Insights in Cholestasis

Wu et al. discuss the mechanisms of fibrosis associated with the development of primary biliary cholangitis (PBC) and primary sclerosing cholangitis (PSC) with special emphasis on cellular changes and relevant signaling pathways (i.e., Notch, Hedgehog, Wnt) in injured cholangiocytes [21]. The overview underpins the notion that impaired bile formation and transport during cholestasis is provoked by extremely complex alterations, limiting treatment options for this condition. Since liver transplantation is presently the only ultimate option to cure progressive cholestatic liver disease, the authors correctly noted that better knowledge on events underlying blockade of bile acid formation and secretion is urgently needed to generate significant diagnostic and therapeutic breakthroughs.

In this regard, Hahn et al. investigated the cytokine IL-13 as a therapeutic target during short term and chronic intrahepatic cholestasis in an *Abcb4*-knockout mouse model, which is a spontaneous model resembling PSC, and further in an *Abcb4*^−/−^/*Il-13*^−/−^ double knockout model [22]. The authors could demonstrate that the loss of IL-13 transiently improves cholestasis and is associated with profound beneficial compositional changes in gut microbiota, as well as the normalization of serum and fecal bile acid composition. The authors, therefore, suggested that targeting *Il-13* expression might be an effective therapeutic means to improve bile duct disorders [22].

## 7. Fibrosis Therapy and Regression

In a non-systematic review, Caligiuri and coworkers used independent electronic sources to identify articles providing information on the molecular mechanisms, underlying the reversal of liver fibrosis [23]. Based on the complexity of the disease, the authors identified a large list of possibilities that were used to attenuate and reverse experimental hepatic fibrosis, including removal of causative agents, clearance/inactivation of activated HSC, modulation of inflammatory processes, increasing ECM degradation, and vascular remodeling [23]. The feasibility and limitations of each intervention in modulating the unfavorable balance between ECM deposition and degradation is critically discussed in this article. In sum, the authors conclude that some of the therapies or drugs could represent promising tools to resolve hepatic fibrosis, but that none of them are already used in human studies or in clinical settings.

One potential drug candidate that is experimentally tested is the cell permeable EX-527, also known as selisistat. This compound is a potent and selective inhibitor of SIRT1, which belongs to the sirtuin family of NAD-dependent HDACs, consisting in mammals of seven proteins (SIRT1-SIRT7). Kundu and colleagues now showed that low doses of EX-527 could alleviate hepatic damage and fibrosis in diabetic Zucker rats by reducing reactive oxygen species formation, the inhibition of inflammation-associated expression of proinflammatory cytokines (i.e., IL-6, IL-1β, TNF-α), and the reducing activities of signaling pathways that contribute to hepatic fibrosis. Furthermore, EX-527 induced the expression of SIRT2, SIRT3, and SIRT4 in the respective model, while SIRT4 but not SIRT2 or SIRT3 expression was lowered in human fibrotic liver tissue. Based on these findings, it is reasonable to conclude that the beneficial effects of EX-527 in the model of obesity-induced hepatic fibrosis are mediated at least in part by the upregulation of SIRT4 [24].

Another very promising anti-fibrotic acting drug was introduced by Reichert et al. In their article, they could show that receptor tyrosine kinase class III inhibitor Crenolanib can improve the recovery from liver fibrosis in rats [25]. In the thioacetamide model and primary HSC cultures, the authors could demonstrate that Crenolanib interferes with HSC proliferation by blocking PDGF-driven signaling pathways, inducing hepatocyte growth factor and fibroblast growth factor-7 and -10, and inducing a stress response associated with inositol-requiring enzyme-1α (IRE1α). This initiates a cell differentiation process that in sum results in an endodermal development of HSC [25]. These findings show that the biological functions of this benzamidazole compound are rather complex and that potential side effects are not fully evaluated yet.

## 8. Conclusions

The current knowledge on how etiological, epigenetic, molecular, and cellular impact progressive fibrosis is enormous. Cell-based models, animal experimentation, and other preclinical studies have identified many fibrotic mediators and pathways that offer a plentitude of potential possibilities to establish antifibrotic therapies. Furthermore, experimental studies have shown that hepatic fibrosis can be indeed improved or even reversed by antioxidants or compounds impacting ECM generation/turnover, the biological properties of liver resident or infiltrating blood cells, or by specifically targeting pro-inflammatory or pro-fibrotic acting pathways. Nevertheless, all these efforts have not identified effective drugs for the treatment of hepatic fibrosis and most clinical trials have shown poor outcomes. This might be due to the fact that progressive fibrosis is more complex than general assumed. Most likely an effective antifibrotic therapy requires the tackling of more than one pathogenic target or pathway. The individual contributions of the Special Issue “*Cellular and Molecular Mechanisms Underlying the Pathogenesis of Hepatic Fibrosis II*” underpin the versatility of potential druggable targets and the hope and limitations associated with them.

## 9. Final Remark

Similar to the first edition of the Special Issue entitled “*Cellular and Molecular Mechanisms Underlying the Pathogenesis of Hepatic Fibrosis*”, published in the year 2020, the second edition received a large number of submissions. Therefore, *Cells* entrusted me as a member of the editorial board to edit another follow up to this Special Issue. The collection of these articles is now open for submission (https://www.mdpi.com/journal/cells/special_issues/93A84S68TE; accessed on 2 August 2022). Research articles, reviews, or shorter perspective articles on all aspects related to this topic are highly welcomed.

## Abbreviations

AP, alkaline phosphatase; Daxx, death-associated protein 6; ECM, extracellular matrix; HDAC(s), histone deacetylase(s); IRE1α, inositol-requiring enzyme-1α; LPS, lipopolysaccharide(s); LSEC, liver sinusoidal endothelial cells; miR(s), microRNA(s); MIF, macrophage migration inhibitory factor; MMP(s), matrix metalloproteinase(s); NAFLD, nonalcoholic fatty liver disease; NASH, nonalcoholic steatohepatitis; PBC, primary biliary cholangitis; PLIN5, perilipin 5; PNPLA3, patatin-like phospholipase domain-containing protein 3; PSC, primary sclerosing cholangitis; RNA, ribonucleic acid; SIRT, sirtuin.
